# Discrimination of Trichosanthis Fructus from Different Geographical Origins Using Near Infrared Spectroscopy Coupled with Chemometric Techniques

**DOI:** 10.3390/molecules24081550

**Published:** 2019-04-19

**Authors:** Liang Xu, Wen Sun, Cui Wu, Yucui Ma, Zhimao Chao

**Affiliations:** 1 Institute of Chinese Materia Medica, China Academy of Chinese Medical Sciences, Beijing 100700, China; xuliang9988@126.com (L.X.); sun.w@outlook.com (W.S.); wucuidalian@163.com (C.W.); yucuim@gmail.com (Y.M.); 2 Storage & Packaging Position, Chinese Materia Medica, China Agriculture Research System, Beijing 100700, China

**Keywords:** near infrared spectroscopy, Trichosanthis Fructus, geographical origin, chemometric techniques, crude drugs, prepared slices, support vector machine-discriminant analysis

## Abstract

Near infrared (NIR) spectroscopy with chemometric techniques was applied to discriminate the geographical origins of crude drugs (i.e., dried ripe fruits of *Trichosanthes kirilowii*) and prepared slices of Trichosanthis Fructus in this work. The crude drug samples (120 batches) from four growing regions (i.e., Shandong, Shanxi, Hebei, and Henan Provinces) were collected, dried, and used and the prepared slice samples (30 batches) were purchased from different drug stores. The raw NIR spectra were acquired and preprocessed with multiplicative scatter correction (MSC). Principal component analysis (PCA) was used to extract relevant information from the spectral data and gave visible cluster trends. Four different classification models, namely *K*-nearest neighbor (KNN), soft independent modeling of class analogy (SIMCA), partial least squares-discriminant analysis (PLS-DA), and support vector machine-discriminant analysis (SVM-DA), were constructed and their performances were compared. The corresponding classification model parameters were optimized by cross-validation (CV). Among the four classification models, SVM-DA model was superior over the other models with a classification accuracy up to 100% for both the calibration set and the prediction set. The optimal SVM-DA model was achieved when C =100, γ = 0.00316, and the number of principal components (PCs) = 6. While PLS-DA model had the classification accuracy of 95% for the calibration set and 98% for the prediction set. The KNN model had a classification accuracy of 92% for the calibration set and 94% for prediction set. The non-linear classification method was superior to the linear ones. Generally, the results demonstrated that the crude drugs from different geographical origins and the crude drugs and prepared slices of Trichosanthis Fructus could be distinguished by NIR spectroscopy coupled with SVM-DA model rapidly, nondestructively, and reliably.

## 1. Introduction

Trichosanthis Fructus, the dried ripe fruits of *Trichosanthes kirilowii* Maxim. or *T. rosthornii* Harms (Fam. Cucurbitaceae), has been commonly used in Traditional Chinese Medicine (TCM) for the treatment of cough with lung heat, sticky phlegm, constipation, thoracic obstruction, and cardiodynia [1]. In modern clinical practice, Trichosanthis Fructus and its TCM prescriptions played a very important role in treating cardiovascular diseases including angina, cardiac failure, myocardial cinfarction, arrhythmia during acute myocardial infarction reperfusion, pulmonary heart disease, and cerebral ischaemic disease [2,3,4]. Because of its high medicinal value and good economic benefit, *T. kirilowii* has been cultivated widely in China [5], such as Shandong, Shanxi, Henan, and Hebei Provinces [6]. Specially, Trichosanthis Fructus produced from Shandong Province, was considered to be genuine since it showed the highest curative effect in traditional clinical use and active constituent content [6,7,8]. However, it was not easy to discriminate the geographical origin by visual inspection. There were a few studies on the discrimination of Trichosanthis Fructus from different cultivars or geographical origins using analytical methods including high pressure liquid chromatography (HPLC) fingerprint [9], seed protein electrophoresis [10], scanning electron microscope [11], and random amplified polymorphic DNA (RAPD), internal transcribed spacer (ITS), and sequence-related amplified polymorphism (SRAP) molecular markers [12,13]. However, these methods were time-consuming, costly, and destructive. Therefore, a fast, accurate, and non-destructive analytical method was established to discriminate the geographical origins of Trichosanthis Fructus in this work.

Near infrared (NIR) spectroscopy is a fast, accurate, and nondestructive technique requiring minimal sample processing before analysis. Coupled with chemometric techniques, it appears to be an effective and powerful analytical tool widely used in different fields, such as agricultural food [14,15], petrochemical [16], pharmaceutical [17], environment [18], metabolomic profiling [19], etc. The NIR region spans the wavelength range between 780 and 2500 nm. The absorption bands in this region correspond mainly to combinations and overtones of the fundamental vibrations of O-H, C-H, S-H, and N-H bonds, which are the primary structural components of organic chemical constituents [20]. As the environment factors including light, climate, water, soil, planting methods, etc. have great influences on the growth quality of the medicinal plants, some chemical constituents of the same crude drug from different geographical origins vary in content. Therefore, NIR spectroscopy has been also used to determine the geographical origins of TCM, such as Radix Pseudostellariae [21], Herba Epimedii [22], and Gastrodiae Rhizoma [23] in recent years. However, there has not been any reports until now on the use of NIR spectroscopy for the discrimination of Trichosanthis Fructus from different geographical origins, and the discrimination between crude drugs and prepared slices. It has been found that the concentrations of total saponins, amino acids and total flavonoids were different in different geographic origins [7,9,24], and the concentrations of 5-hydroxymethylfurfural, vanillic acid, quercetin, luteolin, and sugar in prepared slices showed significant changes compared with crude drugs, especially the concentration of 5-hydroxymethylfurfural increased to nearly 26 times as much as crude drugs of Trichosanthis Fructus (*p* < 0.05 or *p* < 0.01) [25,26,27]. All of the above laid the foundation for the feasibility of the following experiment.

In this study, four chemometric techniques including *K*-nearest neighbors (KNN), soft independent modeling of class analogy (SIMCA), partial least squares discriminant analysis (PLS-DA), and support vector machine discrimination analysis (SVM-DA) were attempted to discriminate Trichosanthis Fructus from different geographical origins. Among them, KNN, SIMCA, and PLS-DA were three linear methods, while SVM-DA was a non-linear method. Principal component analysis (PCA) was conducted on the NIR data to extract some principal components (PCs) as the inputs of the supervised pattern classification models. The number of PCs was optimized by cross-validation.

## 2. Results and Discussion

### 2.1. Spectra Investigation

**Figure 1:**
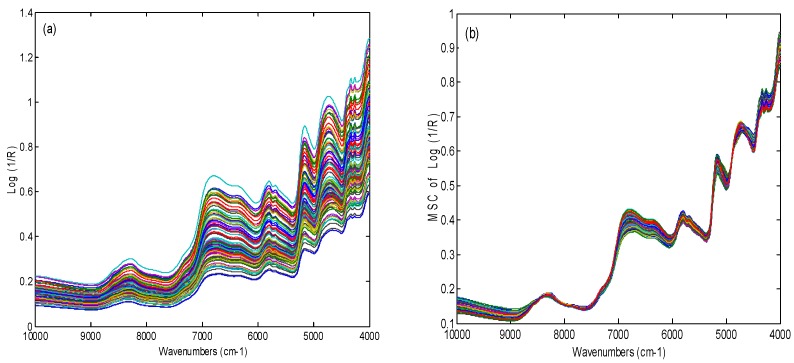
Spectra of Trichosanthis Fructus (**a**) raw data and (**b**) with multiplicative scatter correction (MSC) pretreatment.

The raw NIR spectra of 150 Trichosanthis Fructus samples were shown in Figure 1a. It can be seen that the raw spectra of Trichosanthis Fructus samples from different geographical origins and drug stores were similar. The variation of baseline shifts in spectra was wide, which was attributed to noise, packing density, and particle-size effect. It was difficult to determine specific bands in the raw spectra based on geographical origin because of the high degree of band overlapping. Moreover, the background information and noises contained in the raw spectra could weaken the model performance. Hence, in order to reduce the systematic noise and achieve a reliable model, mathematical spectral preprocessing before calibration was necessary. The multiplicative scatter correction (MSC), as a mathematical transformation method for spectra, was used to remove slope variation and correct scatter effects on the basis of different particle sizes, and correct for additive and multiplicative effects in the spectra. The Savitzky–Golay (SG) filter algorithm could be used to avoid the augmentation of noise which came from the derivatization. Therefore, MSC spectral preprocessing method with SG smoothing was applied in this research and the preprocessing spectra was presented in Figure 1b.

As shown in Figure 1b, ten absorption bands in the spectra could be clearly observed. There was a water absorption band around 5155 cm^−1^ corresponding to the vibration of O-H stretching. There were strong absorption bands belonged to the vibration of C-H stretching (4261 and 4333 cm^−1^), C=O stretching (4673 cm^−1^), -CH_2_ (5797cm^−1^), and N-H stretching (6369 and 6798 cm^−1^). These vibrations were caused by the chemical constituents such as lipids, alkaloids, polysaccharides, free amino acids, proteins, volatile compounds, and so on in Trichosanthis Fructus [28,29,30].

### 2.2. Principal Component Analysis

Principal component analysis (PCA) was a widely used technique for exploring and modeling multivariate data by reducing the dimension of the data matrix and compressing the information into a smaller number of uncorrelated variables called principle components (PCs), which were linear combinations of the original variables [31]. The first principal component, PC1, covered the maximum of the total variance; the second, PC2, was orthogonal to the first one and covered as much of the remaining variation as possible, and so on, until the total variance was accounted for. By plotting the PCs, one could view the interrelationships between different variables and interpret sample patterns, groupings, similarities, and differences. PCA was applied to examine the natural grouping of samples and develop the SIMCA models [32].

**Figure 2:**
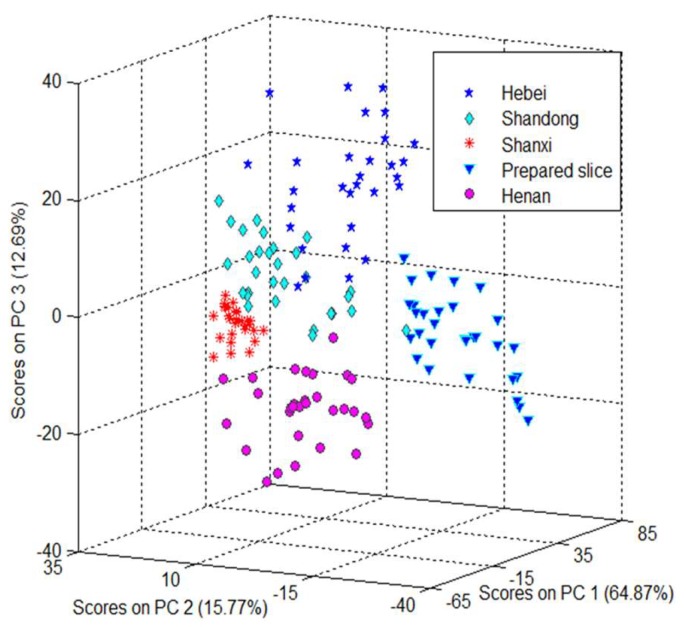
Score cluster plot of the top three principal components (PCs) of Trichosanthis Fructus for the data set.

To visualize the data trends, a score plot was obtained by using the top three principal components (PC1, PC2, and PC3). Figure 2 showed the outcome of the principal component analysis and there were separations in the geographical origins. Figure 2 showed a three-dimensional (3D) space of Trichosanthis Fructus samples represented by PC1, PC2, and PC3. The variance interpreted by PC1, PC2, and PC3 were 64.87%, 15.77%, and 12.69%, respectively. In other words, the top three PCs could load almost the whole NIR spectral information of the samples. There was a clear separation between prepared slices and crude drugs. The crude drugs from different origins were separated roughly. The results indicated that there were differences in the chemical compositions between prepared slices and crude drugs of Trichosanthis Fructus. Though the PCA analysis gave the cluster trend in the 3D space, it could not separate the samples completely. Therefore, effective multivariate classification models were utilized and optimized in the following studies.

### 2.3. Optimation of Models

#### 2.3.1. The Establishment of the KNN Model

**Figure 3:**
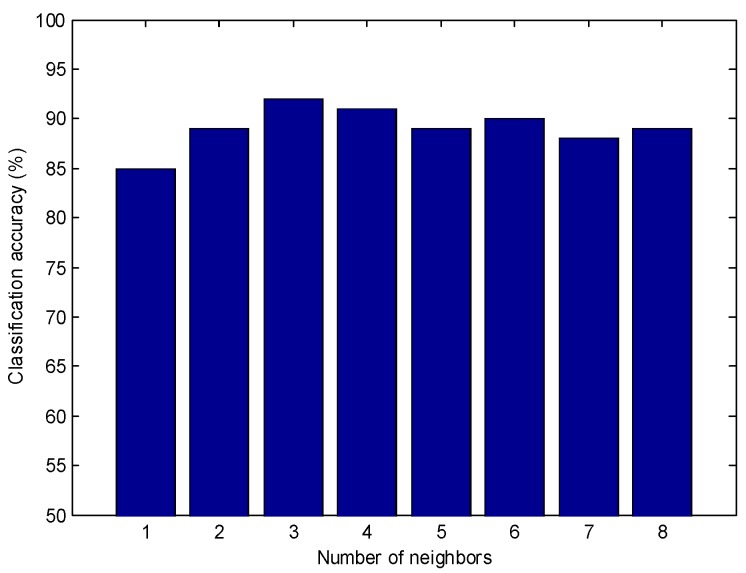
Cross-validation classification accuracy of the *K*-nearest neighbor (KNN) model according to varying parameter *K*.

The KNN method was based on the Euclidean distance between neighbors. Parameter *K*, the number of neighbors which significantly influenced the model performance, could be determined by the classification accuracy (%) for each class. The prediction ability of the model for a given set of *K* values was evaluated by cross-validation, and the *K* value which gave the highest prediction rate was selected as the optimal one. Figure 3 showed the classification accuracy of the KNN model according to different *K* values. The optimal *K* value gave the highest classification accuracy by cross-validation. As shown in Figure 3, eight *K* values (*K* = 1, 2, …, 8) were tested simultaneously for building model and the optimal KNN model was obtained when *K* = 3. The classification accuracy was 92% in the calibration set and 94% in the prediction set, respectively.

#### 2.3.2. The Establishment of SIMCA Model

**Figure 4:**
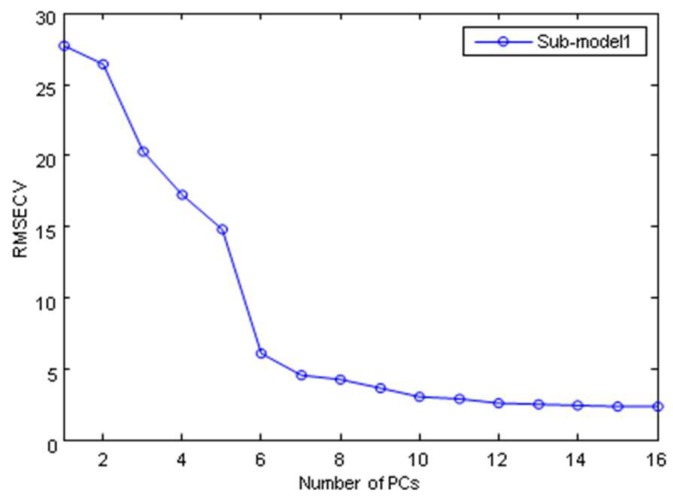
Root mean square error of cross-validation (RMSECV) values according to different number of PCs in sub-model 1.

In SIMCA model, after the initial cluster trends by PCA, the samples were divided into the calibration set and prediction set. Twenty samples were randomly selected from drug stores and each geographical origin as the calibration set, and five sub-models were established. The remaining 50 samples were used for the prediction set to test reliability and stability of each sub-model. The optimal number of the principal components (PCs) for each sub-model was selected based on the root mean square error of cross-validation (RMSECV) by cross-validation. Figure 4 showed an example for the selection of optimal number of PCs in sub-model 1 construction. As shown in Figure 4, most of the improvement in error was achieved before six PCs and the addition of another PC did not greatly lower the RMSECV. These results suggested six PCs for the final sub-model 1. The optimal number of PCs for another four sub-models was determined based on the same criterion. Table 1 listed the optimization and results of SIMCA model. The optimal number of PCs selected for the five sub-models was 6 (Hebei), 4 (Shanxi), 4 (Shandong), 5 (Henan), and 5 (Prepared slices). In the calibration set, the classification accuracy was all above 90% except Shandong and Henan. In the prediction set, the classification accuracies were all 100%, except for Hebei (90%) and Shandong (90%). The average classification accuracy was 93% in the calibration set and 96% in the prediction set. Therefore, NIR spectra combined with SIMCA model had certain feasibility in the discrimination of Trichosanthis Fructus.

#### 2.3.3. The Establishment of the PLS-DA Model

**Figure 5:**
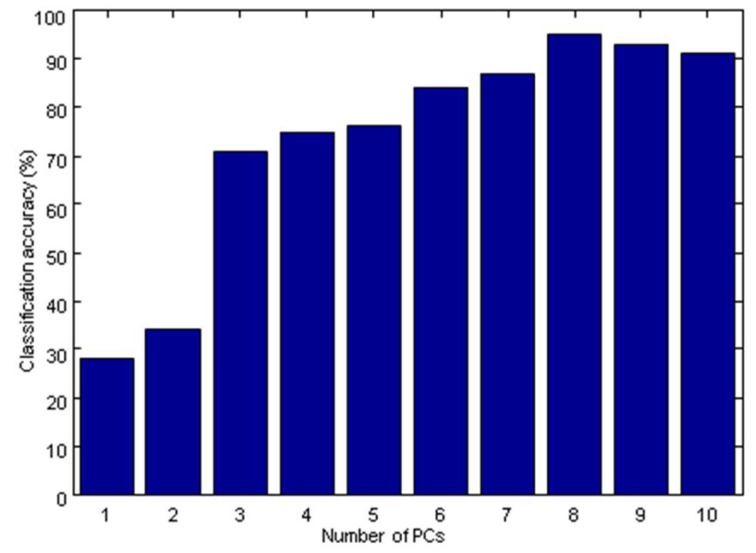
Classification accuracy of the partial least squares-discriminant analysis (PLS-DA) model according to different number of PCs.

Figure 5 showed the classification accuracy according to different number of PCs. The optimal model was obtained when the number of PCs equaled 8 by cross-validation. Its classification accuracy was 95% in the calibration set and 98% in the prediction set.

#### 2.3.4. The Establishment of SVM-DA Model

**Figure 6:**
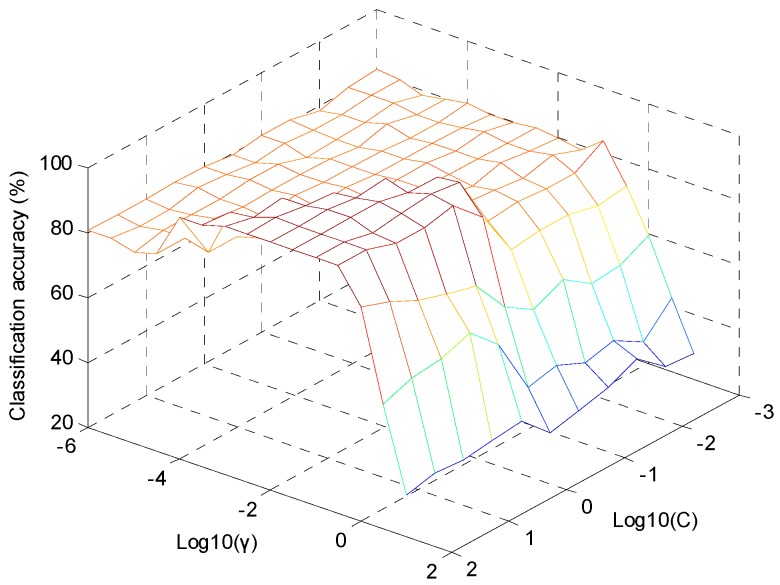
Classification accuracy of the support vector machine-discriminant analysis (SVM-DA) model according to different Log_10_ (γ) and Log_10_ (C) values.

**Figure 7:**
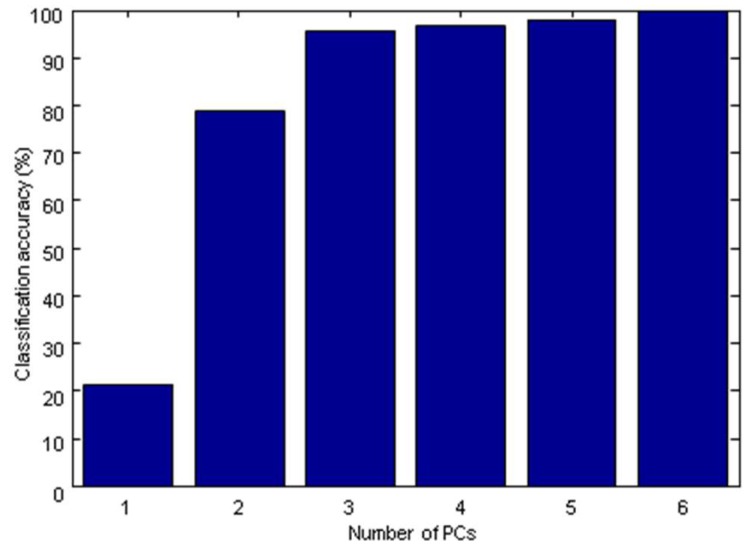
Classification accuracy of the SVM-DA model according to number of PCs.

To obtain the best performance of SVM-DA model, parameters C and γ were optimized by cross-validation. The term C was the penal parameter, which determined the tradeoff between minimizing training error and minimizing model complexity. The γ term was the RBF kernel parameter. In this study, 15 γ values from 10^−6^ to 10 and 11 C values from 10^−3^ to 100 spaced uniformly in log (Log_10_ (γ) = −6.0, −5.5, −5.0, −4.5, …, 1; Log_10_ (C) = −3.0, −2.5, −2.0, −1.5, …, 2) were tested simultaneously for searching the optimal parameter. Figure 6 showed the classification accuracy of the SVM-DA model influenced by values of Log_10_ C and Log_10_ γ. The optimal SVM-DA model was obtained according to the highest classification accuracy by cross-validation. It could be found that the optimal model was achieved when C = 100 and γ = 0.00316 (i.e., Log_10_ (C) = 2, Log_10_ (γ) = −2.5). After parameters C and γ were determined, the optimal number of PCs was obtained according to the highest classification accuracy by 5-fold cross-validation. Figure 7 showed that the highest classification accuracy was achieved when PCs = 6. The classification accuracy in the calibration set and prediction set were both 100%.

### 2.4. Comparison of Four Models

To highlight the good performance in discrimination of Trichosanthis Fructus from different geographical origins, we attempted to compare the performance of 4 classification models of KNN, SIMCA, PLS-DA, and SVM-DA. Table 2 showed the optimal parameters and classification results from the 4 classification models. As shown in Table 2, the classification accuracies of the KNN and SIMCA models did not show better performance than those of PLS-DA model, presumably due to that they put the emphasis more on the similarity within a class. The classification accuracies of SVM-DA gave the best performance compared with KNN, SIMCA, and PLS-DA. The superiority of SVM-DA suggested its superb ability in solving the nonlinear problem in dataset.

## 3. Materials and Methods

### 3.1. Sample Preparation

In this experiment, the fresh ripe fruits of *T. kirilowii* Maxim. were picked up from four geographical origins including Shandong, Shanxi, Hebei, and Henan Provinces (30 samples from each) in October, 2017. They were strung together with their vines, and hung in a cool and drafty room for 6 months. The geographic location of the samples from Shandong Province was Zhuangke Village, Mashan Town, Changqing District, which was acknowledged as the traditional genuine producing area of Trichosanthis Fructus [33]. The crude drugs were obtained after cutting their vines and carpopodium, broke, and smashed. Additionally, 30 batches of prepared slices of uncertain geographical origins were purchased from multiple drug stores from January to March in 2018. The plant materials were identified by Prof. Zhimao Chao (Institute of Chinese Materia Medica, China Academy of Chinese Medical Sciences) as the fruits of *T. kirilowii* Maxim. Voucher specimens were deposited at the 1016 room of Institute of Chinese Materia Medica, China Academy of Chinese Medical Sciences.

Before data acquisition, all samples were dried in a DHG-9053A electric thermostatic drying oven from Shanghai Yiheng Scientific Instrument Co. Ltd. (Shanghai, China) at 60 °C for 4 h to remove moisture. Considering the heterogeneities of the samples, all the samples were crushed into powder by a FW-100 high speed universal grinder from Tianjin Taiste Instrument Co. Ltd. (Tianjin, China). The powder was then screened through a 60-mesh sieve and stored in a glass desiccator for further analysis. After MSC pretreatment, the 150 spectra data were partitioned into a calibration set and validation set, respectively. The former was used to build the calibration model, and the latter was used to test the robustness of the model constructed. About two thirds of the samples were randomly selected to make up the calibration set, and the rest were used as the prediction set. Table 3 shows the details of the tested samples.

### 3.2. Spectral Measurement

The NIR spectra were collected in the diffuse reflectance mode using an MPA multi-purpose FT-NIR spectrometer (Bruker Optics, Ettlingen, Germany) with a Pbs detector and an internal gold background as the reference. Before the sample measurement, the spectrometer needed to be preheated for about 30 min. The spectral data were recorded as the logarithm of the reciprocal reflectance, i.e., log (1/R). Each spectrum was collected by an average of 32 scans performed at 3.857 cm^−1^ interval over the wavelength range of 10,000–4000 cm^−1^. About 5 g of the dried sample powders were densely packed into the sample cup with the loading height of 2 cm. Each sample was collected 3 times in the standard procedure. The average of the 3 spectra collected from the same sample was used in the further analysis. The temperature was controlled at 23 ± 1 °C and the relative humidity at ambient level in the laboratory.

### 3.3. Data Analysis

An OPUS 7.0 from Bruker was used for instrumental and measurement control of the NIR spectrometer as well as for data analysis. The software Solo, version 6.7.1 (Eigenvector Research Inc., Wenatchee, WA, USA) was used for classification methods realization. The NIR spectra (files in MATLAB format, collected by OPUS 7.0) could be recognized by the software for further calculation.

### 3.4. Chemometrics Study

#### 3.4.1. KNN

KNN is a linear and non-parametric supervised pattern recognition method which was first introduced by Fix and Hodges [34]. In this method, distance between the unknown object and each of the objects of the calibration set is determined. The unknown object is classified in the group which the majority of K objects belongs [35]. It is of great importance to select the optimal parameter *K*, which has a great influence on the classification accuracy of the KNN model. The *K* value is optimized by comparing the prediction ability with several *K* values and the one which gives the highest classification accuracy is chosen.

#### 3.4.2. SIMCA

SIMCA is a supervised data classification method based on PCA which was first raised by Wold [36]. In SIMCA, a PCA is performed on each class in the data set and optimal number of PCs is retained to account for most of the variation within each class [37]. Hence, a PCA model is used to represent each class in the data set. The number of PCs retained for each class is important, as retention of too few components can distort the signal or information content contained in the model, whereas retention of too many PCs diminishes the signal-to-noise. RMSECV based on cross-validation is used to find the optimal number of PCs. To perform cross-validation, segments of data are predicted and compared to the actual values, using one, two, three, etc., PCs. The optimal number of PCs is selected when the addition of another PC does not greatly improve the performance of the model.

#### 3.4.3. PLS-DA

PLS-DA is performed in order to find models that allow the maximum separation among classes of objects [38] by hopefully rotating PCA components and to understand which variables carry the class separating information. PLS-DA consists of a classical PLS regression where the response variable is a categorical one (replaced by the set of dummy variables describing the categories) expressing the class membership of the statistical units. Therefore, PLS-DA does not allow for other response variables than the one for defining the groups of individuals. As a consequence, all measured variables play the same role with respect to the class assignment. PLS-DA simultaneously decomposes spectral matrix and class matrix, and extracts the spectral information most related to the classes, which can lead to the establishment of a more accurate classification model [39].

#### 3.4.4. SVM-DA

SVM-DA is a chemometric technique that is originated from binary classification but supports classification of multiple classes [40]. Each classification model is achieved by creating a hyperplane that allows linear separation in the higher dimension feature space, unless the linear boundary in lower dimension input space would accomplish a proper classification. In SVM-DA, this transformation into higher dimensional space is achieved through a kernel function. There exist three classical kernel functions: polynomial kernel function, Gaussian kernel function, and sigmoid kernel function. Selection of kernel function is of great importance on the performance of SVM-DA.

In this work, the popularly used Gaussian kernel function was applied. Its structure was the radial basic function (RBF), also called RBF kernel function. RBF kernel took the form as Equation (1):

(1) $$K\left(x_{i},x_{j}\right)=\exp(-\gamma \|x_{i}-x_{j}\|{}^{2})$$

In order to obtain a good performance of SVM-DA model, two parameters including the penalty parameter (C) and kernel width (γ) in Gaussian kernel function should be optimized. The optimization was achieved by a combination of grid-search approach and 5-fold cross-validation (CV). The optimal C and γ were selected when the highest classification accuracy achieved. After the selection of parameters C and γ, the number of PCs was also optimized based on the highest classification accuracy by CV.

#### 3.4.5. Model Efficiency Estimation

To evaluate the classification performances of the different classification models, the classification accuracy (%) by cross-validation was used as *N_right_*/*N*_0_. *N_right_* and *N*_0_ referred to the number of rightly classified and total number of samples in data set, respectively.

Five-fold CV was used to evaluate the efficiency of classification models. The calibration set was first divided into five subsets of equal size. Sequentially, one subset was tested using the classification model trained on the remaining four subsets. Thus, each instance of the whole calibration set was predicted once so the CV accuracy was the percentage of data.

For SIMCA model construction, RMSECV was used to evaluate the model efficiency based on CV. The RMSECV was defined as Equation (2):

(2) $$RMSECV=\sqrt{\frac{\sum_{i=1}^{n}{\left(y_{i}-{\hat{y}}_{i}\right)}^{2}}{n}}$$

## 4. Conclusions

This study sufficiently demonstrated that NIR spectroscopy coupled with chemometric techniques had high potential to distinguish the crude drugs of Trichosanthis Fructus from different geographical origins and to discriminate the crude drugs and prepared slices in an accurate and non-destructive way. The successful discrimination using chemometric analysis was based on their differences in NIR spectra, which mainly correlated with the differences in their chemical compositions. The differences of crude drugs from different geographic origins might be caused by soil, climate, light, planting methods, and other factors. Light affected the synthesis and accumulation of carbohydrates and nitrogen metabolism of plants, soil affected the absorption of mineral elements in plants, and climate affected the growth cycle of plants resulting in the inconsistency of fruit maturity. All these led to changes in the types and contents of the constituents in Trichosanthis Fructus of different geographic origins. The variations between crude drugs and prepared slices might lie in that the former was only dried from the fresh fruits in the air, while the latter also needed to be steamed through, pressed, shredded, and sun-cured after dried fruits.

Four chemometric techniques (KNN, SIMCA, PLS-DA, and SVM-DA) were applied comparatively to construct the classification models. Among the four classification models, SVM-DA as a non-linear classification method showed superior performance over the linear ones of KNN, SIMCA, and PLS-DA after preprocessing with MSC. The classification accuracy of the calibration set and prediction set were both 100% when C = 100, γ = 0.00316, and PCs = 6. Generally, the non-linear model performed better than the linear models.

The genuineness of herbal medicine depends mostly on its geographical origins. It can be concluded that NIR spectroscopy coupled with chemometric techniques will have more application on the discrimination of TCM according to different geographical origins similarly, which is essential for quality control and traceability management. It also has a promising future to distinguish crude drugs and prepared slices, authenticity, adulteration, and storage period of TCM, all of which cannot be easily recognized by simple visual inspection. Therefore, more representative TCM samples need to be collected and experimented to develop more robust models for prediction in further studies.

## Figures and Tables

**Table 1:** Optimization and results of the soft independent modeling of class analogy (SIMCA) model of Trichosanthis Fructus samples.

Sub-Models	Labels	PCs	Calibration Set		Prediction Set	
			*N_right_*/*N*_0_	CA%	*N_right_*/*N*_0_	CA%
1	Hebei	6	19/20	95	9/10	90
2	Shanxi	4	19/20	95	10/10	100
3	Shandong	4	17/20	85	9/10	90
4	Henan	5	18/20	90	10/10	100
5	Prepared slice	5	20/20	100	10/10	100

**Table 2:** Comparison of the classification accuracy of the *K*-nearest neighbor (KNN), soft independent modeling of class analogy (SIMCA), partial least squares-discriminant analysis (PLS-DA), and support vector machine-discriminant analysis (SVM-DA) models.

Classification Models	Optimal Parameters	Classification Accuracy	
		Calibration Set (%)	Prediction Set (%)
KNN	*K* = 3	92	94
SIMCA	PCs = 6, 4, 4, 5, 5	93	96
PLS-DA	PCs = 8	95	98
SVM-DA	C = 100, γ = 0.00316, PCs = 6	100	100

**Table 3:** A summary of Trichosanthis Fructus samples.

**Sample No.**	**Sample Type**	**Geographic Origins**	Geographic Location *	**Harvesting Time**
1–30	Crude drug	Jinan, Shandong	36°19′ N, 116°19′ E, 127–131 m	Oct 12, 2017
31–60	Crude drug	Anyang, Henan	36°03′ N, 114°23′ E, 68–70 m	Oct 17, 2017
61–90	Crude drug	Anguo, Hebei	38°21′ N, 115°16′ E, 32–33 m	Oct 1, 2017
91–120	Crude drug	Houma, Shanxi	35°19′ N, 111°04′ E, 461–466 m	Oct 4, 2017
121–150	Prepared slices	Uncertain	Uncertain	Jan-Mar, 2018

* Geographic location is marked in the order of latitude, longitude, and altitude.
